# Artesunate attenuates inflammation and neurological impairment in intracerebral hemorrhage mice by inhibiting microglia NLRP3/ASC/Caspase-1

**DOI:** 10.1590/1414-431X2026e14465

**Published:** 2026-10-30

**Authors:** Qiang Luo, Yuan Chai, Geng Qin, Yiwen Ma, Zhixiong Zhou, Xiaohuan Huang, Junxiu Xi, Yu Miao

**Affiliations:** 1 Department of Neurosurgery, Xi'an Central Hospital, Xi'an Jiaotong University, Xi'an, China

**Keywords:** ART, ICH, NLRP3, Pyroptosis, Microglia, Inflammation

## Abstract

Intracerebral hemorrhage (ICH) impairs brain function through several mechanisms, including local inflammation, which exacerbates neuronal injury. Given the complex pathology of ICH, understanding how to mitigate these effects is essential for improving recovery outcomes. While artesunate (ART) has been recognized for its neuroprotective effects, its role in neural recovery following ICH remains unclear. This study aimed to address this gap by investigating the potential neuroprotective role of ART in ICH recovery. We employed a male C57BL/6 mouse model of ICH and administered ART post-injury. Brain samples were collected three days later for analysis. Hematoxylin and eosin staining was used to assess localized damage, and brain edema was measured using the dry/wet weight method. Neurological function was evaluated through the modified neurological severity score (mNSS) and the corner test. Local inflammation was assessed by measuring interleukin (IL)-1β, tumor necrosis factor (TNF)-α, and myeloperoxidase (MPO). NLRP3/ASC/Caspase-1 expression was analyzed via western blotting to evaluate pyroptosis. Immunofluorescence analysis revealed that microglia predominantly undergo pyroptosis. *In vitro*, ART inhibited NLRP3-mediated pyroptosis in microglia and reduced the secretion of IL-1β and IL-18. Ultimately, ART decreased neuronal apoptosis, promoted neurogenesis, and improved neurological function after ICH. Our findings suggest that ART may alleviate ICH damage by downregulating NLRP3-mediated microglial pyroptosis, reducing neuronal apoptosis, and decreasing inflammation, offering promising potential for improved ICH treatment and prevention.

## Introduction

Intracerebral hemorrhage (ICH) is a severe and often fatal type of stroke, accounting for approximately 30% of all stroke cases (1). Its high mortality and morbidity rates make it a critical area of concern. When a blood vessel ruptures suddenly in the brain, it creates a hematoma that causes mechanical damage to surrounding tissues and leads to a rapid increase in intracranial pressure, which is considered primary brain injury (2). Secondary brain injury then follows, driven by a range of pathological processes triggered by the breakdown products of red blood cells. These processes include inflammatory responses, mitochondrial dysfunction, oxidative stress, neuronal apoptosis, and disruption of the blood-brain barrier (3). Open surgery has been shown to offer little benefit to ICH patients. Although recent studies suggest that minimally invasive surgical approaches may provide new opportunities for ICH intervention, their applicability still requires further investigation (4). Consequently, current research is focusing on understanding the mechanisms underlying secondary injury in ICH, with the aim of providing new theoretical foundations for clinical interventions.

As a water-soluble derivative of artemisinin, artesunate (ART) is characterized by high efficiency, low toxicity, and good tolerance in anti-malaria treatments (5). The World Health Organization (WHO) recommends ART as a first-line drug for cerebral malaria and other severe forms of malaria (6). Beyond its antiparasitic effects, ART also exerts anti-inflammatory, anti-tumor, and anti-microbial effects (7). Its mechanisms are diverse, including the inhibition of production of inflammatory factors, induction of interleukin, promotion of iron-dependent apoptosis, and inhibition of angiogenesis (8). These findings collectively demonstrate that ART is a multi-functional drug with potential applications in diseases involving various pathophysiological mechanisms, particularly brain diseases. Recent studies have also shown that ART can reduce neuronal apoptosis in malaria-infected mice, not by targeting the malaria parasite directly but by offering neuroprotective effects to the neurons themselves (9). Furthermore, it has been proven that ART preserves neurons after traumatic brain injury and promotes the release of neurotrophic factors such as BDNF, GDNF, and NT-3, ultimately improving neurological function scores in experimental rats (10). These studies suggest that ART may play a significant role in protecting neural function following ICH.

Research has confirmed that ART can mitigate secondary damage from ICH by promoting ferroptosis in microglia and reducing inflammatory responses (11). The role of NOD-like receptor family pyrin domain containing 3 (NLRP3)-mediated pyroptosis in microglia has been established as a critical component of neuroinflammation following ICH (12). However, the effects of ART on pyroptosis in microglia following ICH remain unclear. We hypothesized that ART would alleviate pyroptosis in microglia after ICH and improve neurological recovery. To test this hypothesis, we investigated inflammation levels, neuronal death, and neurological function after ICH using HE staining, immunostaining, NeuN/TUNEL double-labeling staining, western blot, and behavioral tests.

## Material and Methods

### Animals

C57BL/6 mice, aged 6 to 8 weeks and weighing 20 to 25 grams, were obtained from the Beijing Vital River Experimental Animal Centre (China). Only male mice were used in this study. The animals had *ad libitum* access to food and water and were maintained on a 12-h light/dark cycle. This study was approved by the Ethics Committee of Xi'an Jiaotong University, and all animal experiments were authorized by the Ethics Committee of Xi'an Central Hospital, in accordance with the guidelines set by the China Council on Animal Care.

### ICH modeling and ART treatment

The ICH model was established based on previous literature (13). In this study, male C57BL/6 mice were anesthetized using 4% isoflurane (induction) and maintained with 1-2% isoflurane. The mice were then positioned in a stereotaxic frame for surgical procedures. A 0.6-mm diameter burr hole was drilled at the following coordinates: 0.8 mm anterior to the bregma and 2.0 mm lateral to the midline. In the ICH group, 0.075 U (0.1 U/µL) collagenase VII (Sigma, USA) was injected at a rate of 0.1 µL/min into the basal ganglia at a depth of 3.5 mm. The syringe was left in place for 10 min post-injection to prevent backflow. In the Sham group, mice underwent the same surgical procedure, but instead of collagenase, 0.75 µL saline was injected. In the ICH+ART group, mice received intraperitoneal administration of 150 mg/kg per day artesunate (ART, Sigma) for three days post-ICH. All animal procedures were performed by trained personnel to minimize discomfort and stress during the experiment. After surgery, food was provided on the cage floor, and mice were kept warm until they fully recovered.

### Cell culture and treatment

The BV-2 microglial cell line was obtained from Procell (China) and cultured in complete medium supplemented with 10% FBS and DMEM, under a humidified 5% CO_2_ atmosphere. BV-2 cells were randomly assigned to one of four groups: the control group, which received no treatment; the thrombin (TM) group, treated with thrombin (40 U/mL, Sigma) for 12 h; the TM+ART group, treated with both thrombin and ART (25 µg/mL); and the TM+vehicle group, treated with thrombin and an equal volume of DMSO.

### Western blotting

Mice from each group were rapidly decapitated following anesthesia, and the striatum was carefully isolated on ice after skull dissection. Striatal tissue or cultured BV-2 cells were homogenized on ice with 150 µL of RIPA lysis buffer. The homogenate was then centrifuged at 12,880 *g* for 30 min at 4°C. The protein concentration in the supernatant was determined using a NanoDrop spectrophotometer (Thermo Fisher Scientific, USA). The protein solution was mixed with loading buffer at a 4:1 ratio, heated at 100°C for 10 min, and stored at -80°C. For western blot analysis, 25 µg of protein from each sample was separated using 8-12% SDS-PAGE gel and transferred to 0.45-µm PVDF membranes. The membranes were blocked with 5% bovine serum albumin (BSA) for 1 h at 37°C and then incubated overnight at 4°C with primary antibodies against tumor necrosis factor (TNF)-α (ab183218, 1:2000), IL-1β (ab254360, 1:1000), MPO (ab208670, 1:1000), NLRP3 (ab263899, 1:1000), ASC (ab283684, 1:1000), Caspase-1 (ab138483, 1:1000), GSDMD (ab219800, 1:1000), NGF (ab6199, 1:1000), BDNF (ab108319, 1:1000), and β-actin (ab8227, 1:3000). After washing the membranes three times with TBST, they were incubated for 1 h with goat anti-rabbit IgG conjugated to horseradish peroxidase (HRP) (Beyotime, China). Protein bands were visualized using a chemiluminescence ECL Western Blotting Substrate Kit (Merck-Millipore, USA) according to the manufacturer's instructions. The optical density of the protein bands was quantified in arbitrary densitometry units using ImageJ software (NIH, USA). Protein levels were normalized to β-actin as a loading control, and the intensity of each protein band was compared to the control group for analysis.

### H&E staining

The brains were isolated and sectioned into 4-mm coronal slices. For histopathological analysis, tissue slices were fixed in 10% formalin at room temperature for 24 h, embedded in paraffin, and then cut into 4-µm sections. The sections were deparaffinized and stained with hematoxylin and eosin (H&E) at room temperature for 10-15 min. The stained sections were examined using an Olympus microscope (Japan).

### Immunohistochemistry

Mice were anesthetized and brains were removed and sectioned into 15-μm coronal slices using a sliding freezing microtome. For BV-2 cells, 1×10^5^ cells were fixed on single chamber slices with 95% ethanol. Sections and slices were pretreated with Triton X-100, goat serum, and PBS at 37°C for 100 min, then incubated overnight at 4°C with antibodies against IBA-1 (ab283319, 1:100) and NLRP3 (ab270449, 1:100). The secondary antibodies (Abcam, USA) were applied at room temperature for 1 h. After washing with PBS, DAPI was added for nuclear staining. The slices were mounted and imaged using a Nikon A1R confocal microscope (Nikon, Japan). Fluorescence data were analyzed with ImageJ software (NIH, USA).

For bright-field staining, sections were first quenched with a peroxidase solution (3% H_2_O_2_, 10% methanol in PBS) for 15 min before blocking. After incubation with the primary antibody against ASC (ab307560, 1:1000), NLRP3 (ab270449, 1:100), and Caspase-1 (ab138483, 1:1000), sections were treated with biotinylated secondary antibodies (Abcam) for 2 h. Signal enhancement was achieved using an avidin-biotin kit (Vector Laboratories, USA) for 1 h, and the staining was visualized with 3,3-diaminobenzidine (Vector Laboratories).

### NeuN/TUNEL immunofluorescent double-labeling staining

Briefly, double staining of the neuron marker NeuN (ab104224, 1:1000) and TUNEL were conducted using an *in situ* Apoptosis Detection Kit (Sigma) according to the manufacturer's instructions. The sections were then mounted and imaged using a Nikon A1R confocal microscope.

### ELISA

The concentration of interleukin 1 beta (IL-1β) and interleukin 18 (IL-18) in microglia supernatant was measured using the ELISA Kit (Mlbio, China), following the provided instructions. Absorbance was read at 450 nm using a plate reader (Flexstation3, Molecular Devices, USA).

### Analysis of brain edema

Brain water content was measured using the wet/dry weight method. After ICH modeling, brains were quickly removed and placed individually in pre-weighed aluminum foil. The samples were then dried in an oven at 90°C for 72 h and reweighed to determine the dry weight. The percent moisture content was calculated using the formula: moisture content (%) = wet weight - dry weight / wet weight × 100%.

### mNSS score

Three days postoperatively, the revised modified neurological severity score (mNSS) was used to evaluate motor, sensory, and reflex functions in ICH mice through ten tests as described previously (14). Each test was scored as follows: zero for normal function, one for moderate impairment, and two for complete impairment. The maximum possible score was 18 points, with higher scores indicating more severe nerve injury.

### Corner test

Each mouse was placed in a 30-degree corner formed by two 20-cm glass boards, positioned halfway into the corner as described previously (15). The number of left and right turns made by each mouse as it moved deeper into the corner was recorded. After 15 trials per mouse, the percentage of right turns was calculated.

### Morris water maze

The Morris water maze test was performed as described previously (16). The maze consisted of a circular pool (120 cm diameter, 40 cm height) divided into four quadrants, filled with water maintained at 20-22°C. A dark grey escape platform was placed in one quadrant. Six days before inducing ICH, mice underwent training for six days, completing four trials per day with a one-hour interval between trials. During each trial, mice had up to one minute to locate the platform and could remain on it for a maximum of 30 s. Three days after ICH, a probe trial was conducted in which the platform was removed. Mice were placed in the quadrant opposite the target quadrant and allowed to swim for 60 s. Key parameters recorded included the number of crossings over the former platform location, the total time spent in the target quadrant, latency to first crossing the platform area, and swimming speed. These measures were used to assess spatial learning and memory. Each trial lasted one minute, and data analysis was performed using SMART v3.0 software (Panlab, USA).

### Novel object recognition (NOR) test

The NOR test was performed on day 7 post-ICH as described previously (17). On day 1, mice were allowed to habituate to an open field (47×26×20 cm) for 5 min. On day 2, they were introduced to two identical novel objects (green cubes, 4×4×3 cm) for 10 min. After a one-hour interval, mice were exposed to one novel object (white ball, 5 cm in diameter) and one familiar object (green cube) for 5 min. To assess recognition memory, the discrimination index was calculated as the ratio of time spent with the novel object to the total time spent exploring both objects. Exploration was defined as any direct contact with the object using the mouth, nose, or paws, or when the nose was within 0.5 cm of the object. Incidental contacts, as well as behaviors such as standing, sitting, or leaning on the object, were not counted as exploration.

### Statistics

Statistical comparisons were performed using Prism 8 software (GraphPad Software Inc., USA), and detailed information is provided in the figure legends. All data are reported as means±SE. An unpaired *t*-test was used to assess differences between two groups, and ANOVA followed by Tukey's multiple comparisons test or Games-Howell's multiple comparisons test was used for comparisons of three or more groups. Statistical significance was set as P<0.05.

## Results

### ART reduced brain inflammation and neurological damage post-ICH

To assess the protective effect of ART on ICH, we selected 3 days post-ICH as the observation point. As shown in the striatum HE staining, postoperative ART administration effectively attenuated ICH-induced tissue edema and fragmentation (Figure 1A). To further evaluate whether ART-induced tissue changes resulted in functional improvements, we first performed mNSS scoring on each group, which revealed that ART significantly mitigated neurological impairment in mice 3 days post-ICH (Figure 1B). Given that the early inflammatory response following ICH is a major signature of injury, and as ART has been reported to have favorable inflammatory modulatory effects, we performed immunoblotting assays on the striatum 3 days post-ICH. The results showed that the expression of inflammatory markers MPO, TNF-α, and IL-1β was significantly elevated 3 days post-ICH, whereas ART reduced the inflammatory response following ICH (Figure 1C and D). Brain edema, a critical risk factor for injury exacerbation, was addressed in the previous HE staining. To further evaluate the effect of ART, we examined brain water content in different groups, and the results demonstrated that ART was effective in reducing brain edema after ICH (Figure 1E), leading to recovery of neurological function (Figure 1F).

**Figure 1:**
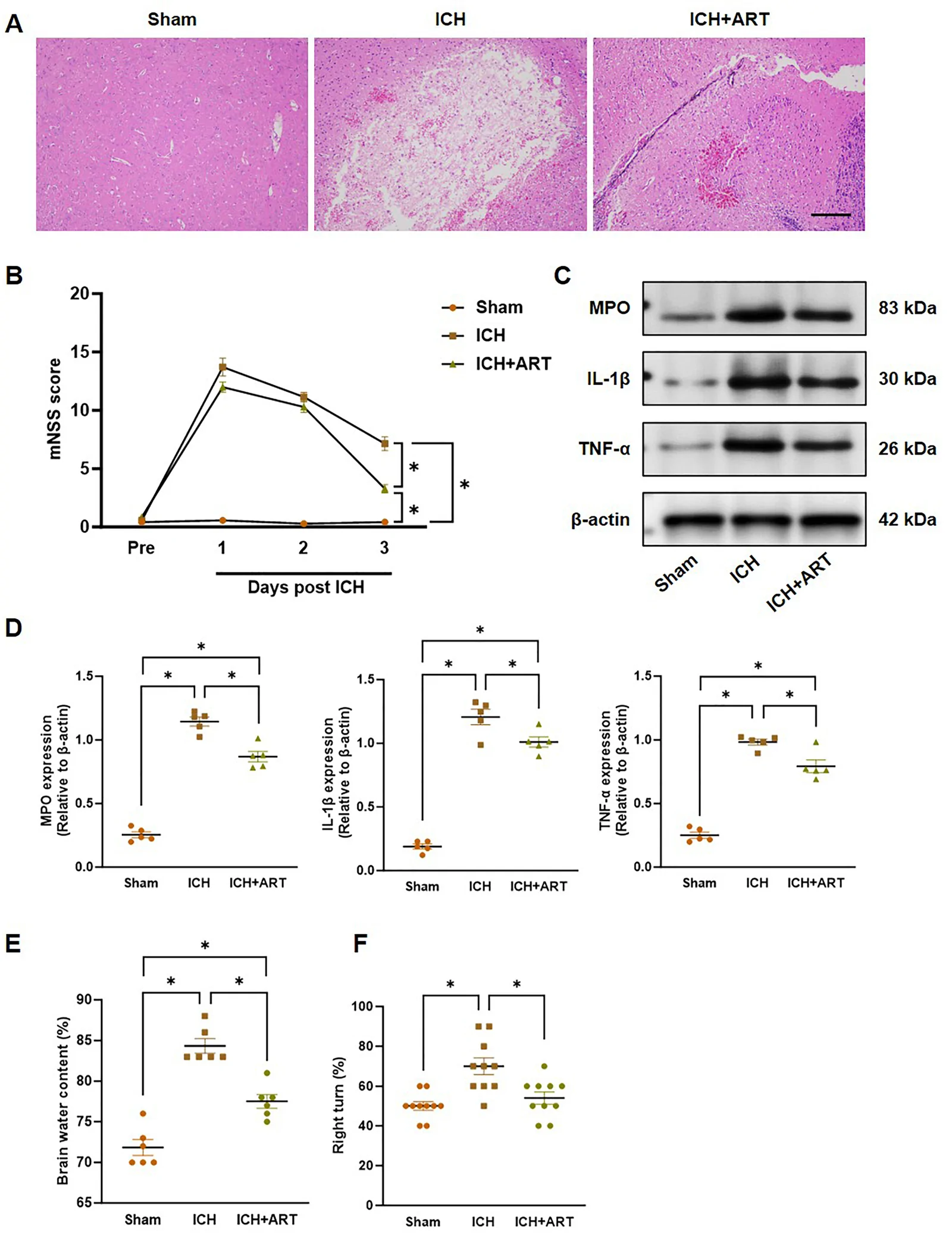
Artesunate (ART) ameliorated mice neuroinflammation and neurological lesions at 3 days post-intracerebral hemorrhage (ICH). **A**, Representative images of striatum HE staining from mice in sham group, ICH group, and ICH+ART group (scale bar=100 μm). **B**, Modified neurological severity scores (mNSS). **C** and **D**, Western blot bands and bar graphs demonstrating the protein expression of myeloperoxidase (MPO), interleukin (IL)-1β, and tumor necrosis factor (TNF)-α in the striatum. **E**, Bar graph demonstrating the brain water content of mice. **F**, Bar graph showing the percentage of right turns in the corner test of mice. Data are reported as means±SE; n=5-10 mice/group. *P<0.05 (ANOVA followed by Tukey's multiple comparisons test).

### ART attenuated microglial NLRP3/ASC/Caspase-1 signaling post-ICH

To investigate whether ART affects cellular pyroptosis after ICH, we first performed immunohistochemical staining for ASC, NLRP3, and Caspase-1, and quantified the number of positive cells. The results revealed that the number of positive cells for ASC, NLRP3, and Caspase-1 significantly increased after ICH, while ART administration significantly reduced the number of positive cells (Figure 2A-F). These findings suggest that the protective effect of ART may be associated with the NLRP3/ASC/Caspase-1 signaling pathway. To identify the specific target cells of ART, we performed immunofluorescence double-labeling staining for NLRP3 and IBA-1. The images showed a significant increase in the number of microglia at 3 days post-ICH, which was reduced following ART administration (Figure 2G and H), further supporting the immunomodulatory effect of ART identified earlier. Additionally, when analyzing the intensity of NLRP3 in IBA-1 positive cells, we found that ART significantly decreased NLRP3 expression in microglia post-ICH (Figure 2I), suggesting that ART may exert its immunomodulatory effects through the regulation of microglial NLRP3 expression.

**Figure 2:**
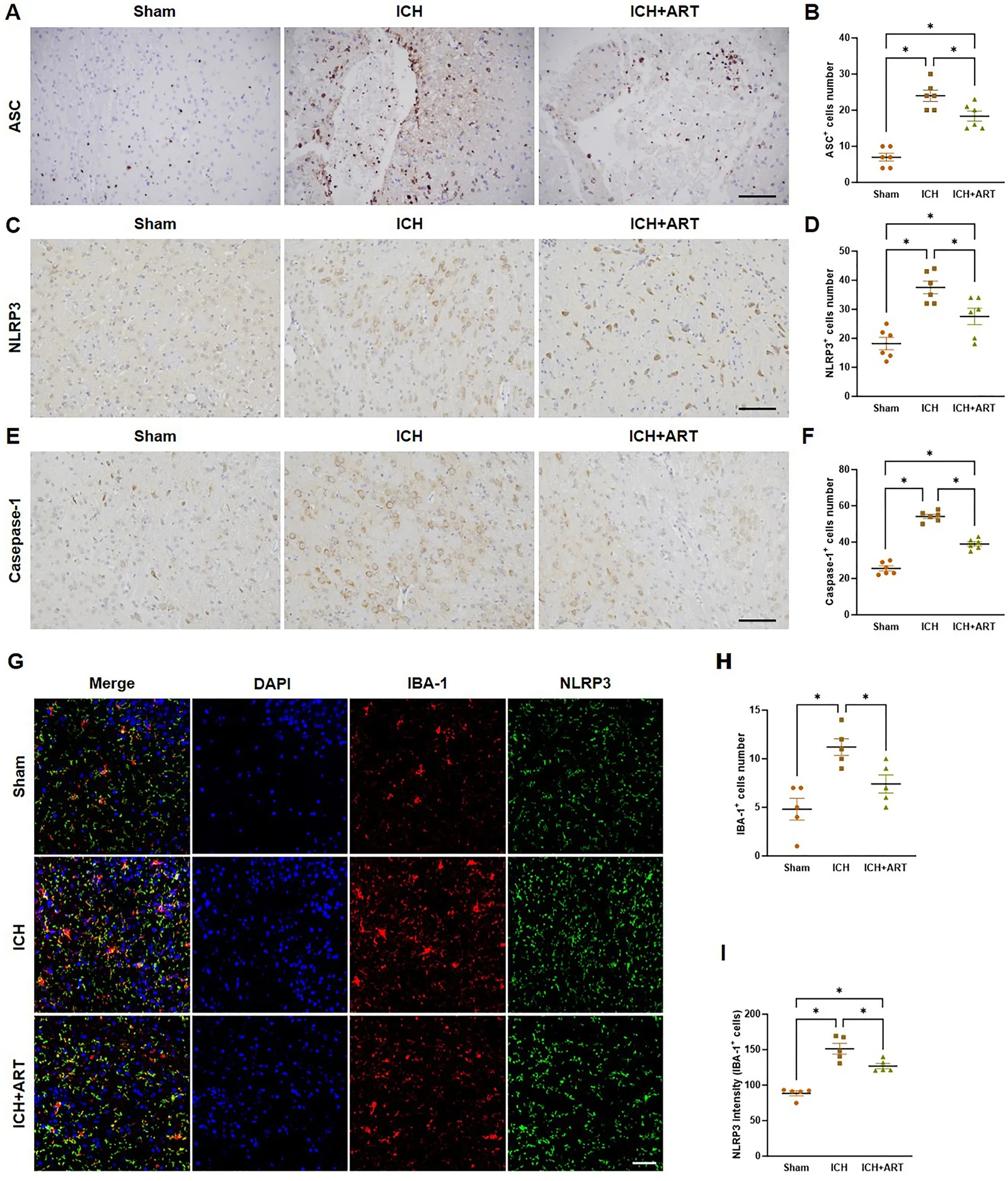
Artesunate (ART) suppressed the NLRP3/ASC/Caspase-1 pathway activated in microglia post-intracerebral hemorrhage (ICH). **A**-**F**, Representative images and bar graphs demonstrating the NLRP3/ASC/Caspase-1 expression and positive cells numbers in the striatum of the sham group, ICH group, and ICH+ART group at 3 days post-ICH. **G**-**I**, Immunofluorescence images of the striatum of each group: IBA-1 (red staining) and NLRP3 (green); scale bar=50 μm. The bar graphs present the quantification of the IBA-1 positive cells numbers and NLRP3 staining intensity in IBA-1-positive cells. Data are reported as means±SE; n=5 mice/group. *P<0.05 (ANOVA followed by Tukey's multiple comparisons test).

### ART alleviated thrombin-induced microglia pyroptosis *in vitro*

In the previous section, we demonstrated that ART attenuated microglial NLRP3/ASC/Caspase-1 signaling, which is an important pathway involved in pyroptosis. However, whether ART affects microglial pyroptosis specifically remains unexplored. To address this, we stimulated microglia with thrombin and examined NLRP3/ASC/Caspase-1 signaling along with GSDMD, a key protein associated with pyroptosis. The results showed a significant increase in pyroptosis in thrombin-stimulated microglia, which was accompanied by activation of the NLRP3/ASC/Caspase-1 signaling pathway. ART, however, significantly alleviated this response (Figure 3A and B). To further validate these findings, we performed immunofluorescence, which confirmed the immunoblotting results. Specifically, thrombin significantly elevated NLRP3 levels in cultured BV-2 cells, while ART effectively reversed this effect (Figure 3C and D). Microglial pyroptosis is notably important for the release of inflammatory factors. To assess ART's role in reversing microglial pyroptosis, we measured the levels of IL-18 and IL-1β in different groups using ELISA. The results showed that thrombin significantly increased the release of IL-18 and IL-1β from microglia, while ART significantly reduced the levels of these inflammatory factors (Figure 3E and F).

**Figure 3:**
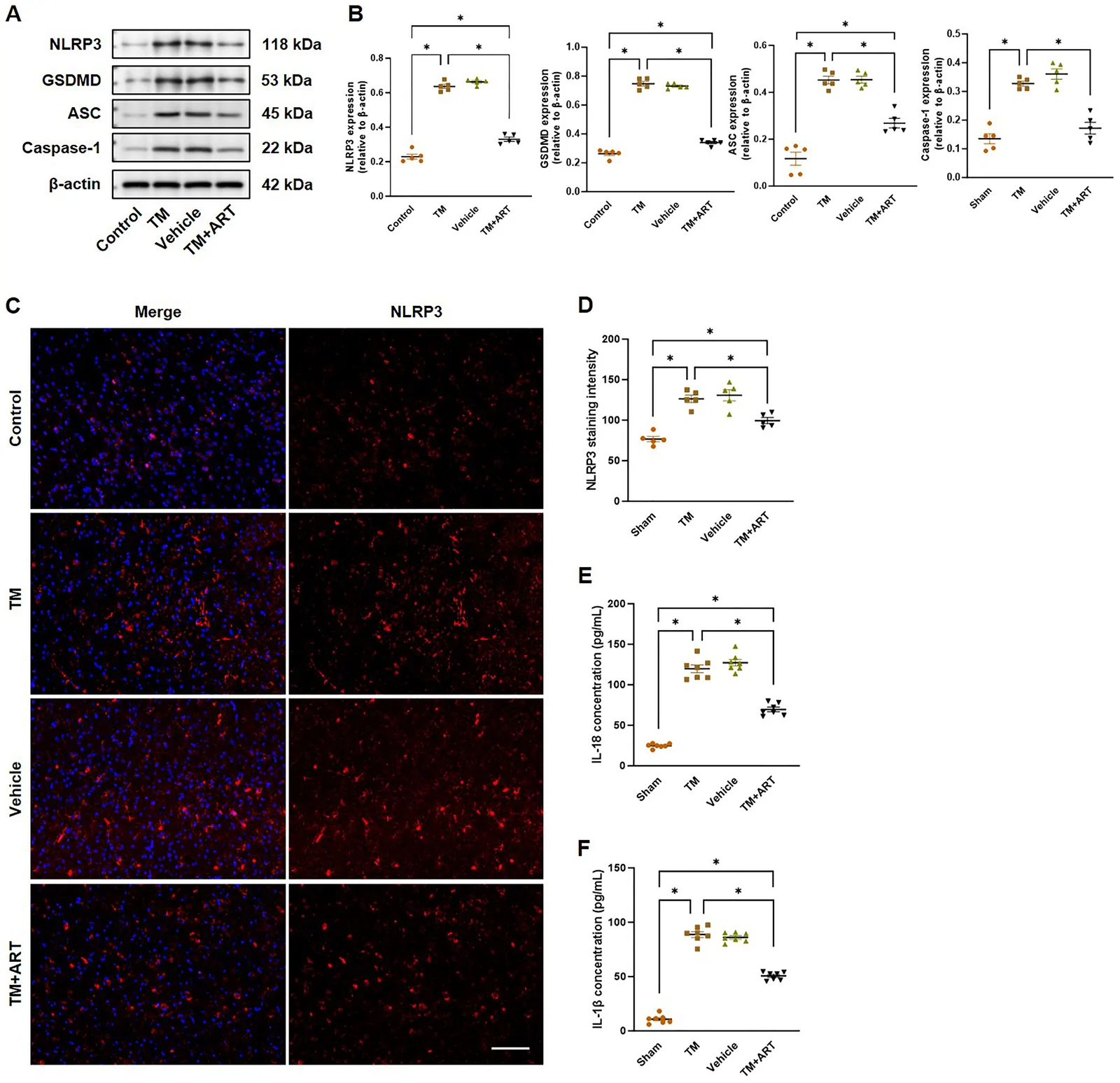
Artesunate (ART) suppressed thrombin (TM)-induced microglia pyroptosis and the release of pro-inflammation factors *in vitro* in mice post-intracerebral hemorrhage (ICH). **A** and **B**, Western blot bands and bar graphs demonstrating the protein expression of NLRP3, GSDMD, ASC, and Caspase-1, in cultured BV-2 in the control group, thrombin group, thrombin+vehicle group, and thrombin+ART group. **C** and **D**, Representative immunofluorescence images of each group: NLRP3 (red staining); scale bar=50 μm. The bar graph shows the NLRP3 staining intensity. **E** and **F**, Supernatant concentration of interleukin (IL)-18 and IL-1β in each group. Data are reported as means±SE; n=5 repetitions/group. *P<0.05 (ANOVA followed by Tukey's multiple comparisons test).

### ART reduced neurological impairment post-ICH

In previous results, we initially observed the effect of ART on neurological recovery after ICH. To further confirm this, we first performed immunoblotting to assess the expression of neurological function-related factors, NGF and BDNF, in the ipsilateral cerebral hemisphere. The results showed that the expression of nerve growth factor (NGF) and brain-derived neurotrophic factor (BDNF) was significantly decreased post-ICH, but this decline was effectively mitigated by ART treatment (Figure 4A and B). Additionally, NeuN/TUNEL staining of the ipsilateral striatum further supported these findings. By analyzing the number of double-labeled cells, we demonstrated a significant increase in striatal neuron apoptosis following ICH, which was remarkably reduced by ART administration (Figure 4C and D). Neurological dysfunction after ICH is often associated with cognitive decline. To assess whether the neuroprotective effects of ART could influence cognitive changes post-ICH, we conducted the Morris water maze and NOR tests. The results showed that the number of platform crossings was significantly reduced in ICH mice, but ART administration led to an improvement in platform crossings (Figure 4E and F). In the NOR task, ICH mice spent significantly less time exploring the novel object, whereas ART treatment improved this behavior (Figure 4G).

**Figure 4:**
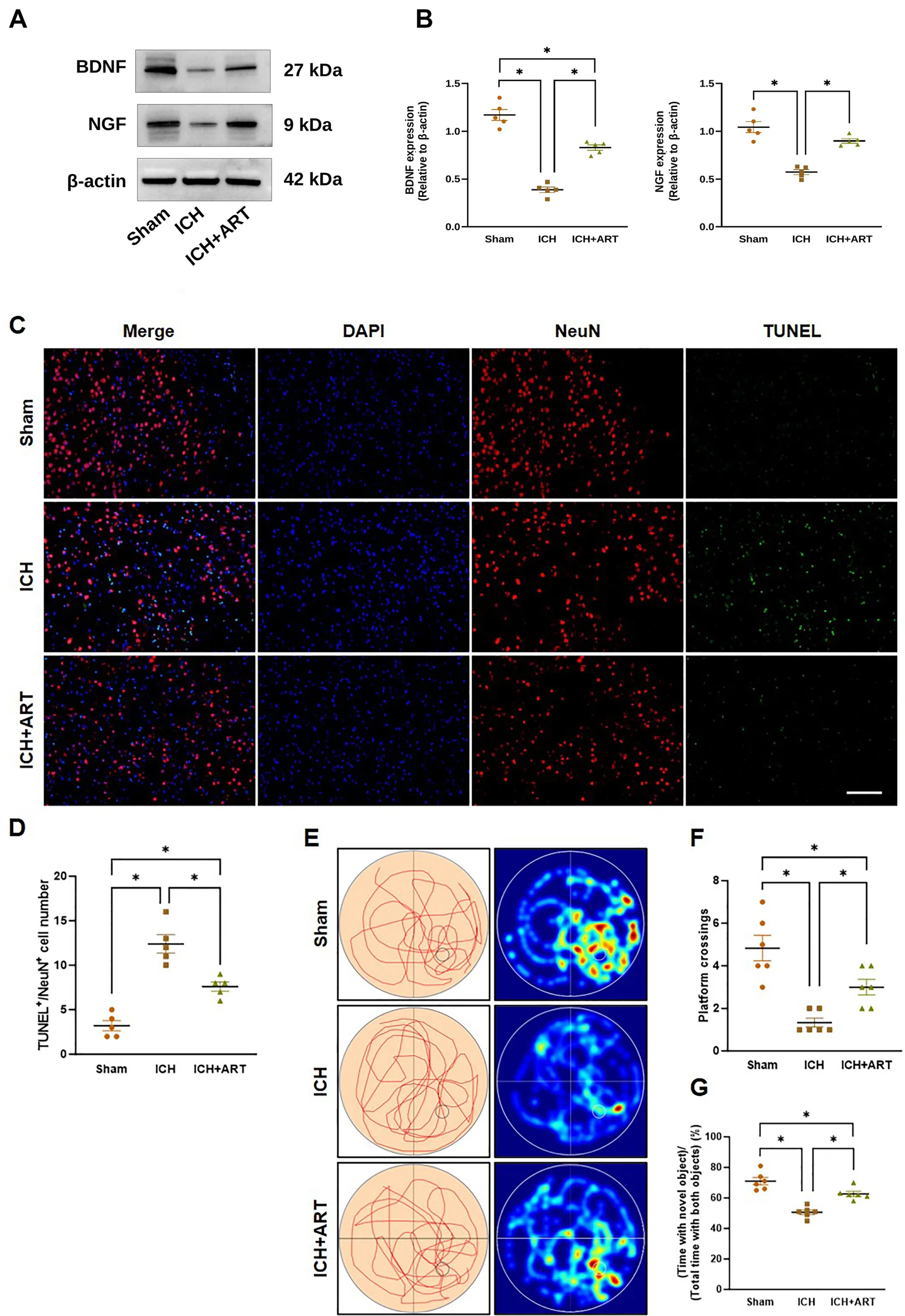
Artesunate (ART) reduced neurological damage and alleviated cognitive impairment in mice post-intracerebral hemorrhage (ICH). **A** and **B**, Western blot bands and bar graph demonstrating the protein expression of BDNF and NGF in ipsilateral cerebral hemispheres in each group. **C** and **D**, Immunofluorescence images of the ipsilateral striatum of each group: TUNEL (green staining) and NeuN (red); scale bar=50 μm. The bar graph shows the quantification of the TUNEL and NeuN co-labeling cells in each group. **E**-**G**, Images and bar graphs show the results of the Morris water maze and novel object recognition (NOR) test of mice, evaluated by the number of platform crossings and novel object exploring time. Data are reported as means±SE; n=5-6 mice/group. *P<0.05 (ANOVA followed by Tukey's multiple comparisons test).

## Discussion

ICH is a common and devastating condition that often leads to severe neurological deficits or even death, placing a heavy burden on individuals, families, and society. The inflammatory response following brain hemorrhage is a major factor driving disease progression. During this process, the recruitment of microglia and the release of pro-inflammatory cytokines play crucial roles. Additionally, peripheral inflammatory cells, such as leukocytes, can infiltrate the central nervous system through the damaged blood-brain barrier, further exacerbating the inflammatory damage and worsening the condition (18). Therefore, targeting the reduction of inflammatory infiltration and the inhibition of the inflammatory cascade following cerebral hemorrhage is essential for developing new therapeutic approaches and strategies, which holds significant clinical and social importance.

ART has been extensively studied for its potential in antitumor therapy, prevention of organ damage and dysfunction, immune and inflammatory modulation, neurotransmission regulation, and treatment of type I diabetes (19). The WHO malaria treatment guidelines recommend ART as a first-line treatment for cerebral malaria and other severe forms of malaria, with no toxicity observed at non-intestinal doses ranging from 1.75 to 4 mg/kg (20). Moreover, ART is relatively inexpensive, making it an attractive option. As the most promising artemisinin derivative and the only water-soluble form currently in clinical use, ART benefits from high blood-brain barrier permeability, which has sparked growing interest in its research for central nervous system applications.

Previous studies have demonstrated that ART protects the blood-brain barrier in mice with subarachnoid hemorrhage (SAH) by activating the sphingosine 1-phosphate receptor 1/phosphatidylinositol 3-kinase (S1pR1/PI3K) signaling pathway (21). Preliminary *in vitro* studies have shown that ART promotes the proliferation of neural stem cells via the PI3K/Akt signaling pathway (22). Additionally, several studies have reported the protective effects of ART after cerebral hemorrhage, including activation of Nrf2 and ROS-dependent p38 MAPK pathways to inhibit oxidative stress and inflammation, thereby preventing cerebral ischemia-reperfusion injury (23). ART has also been shown to exert neuroprotective effects against cerebral ischemia-reperfusion injury in rats via the TLR-4/NF-κB pathway (24). However, to date, no studies have explored the involvement of ART in the NLRP3 inflammasome.

NLRP3 inflammasomes, comprising NLRP3 receptor proteins, ASC-linked proteins, and Caspase-1 effector proteins, are crucial components of the innate immune system. Pathogen-associated molecular patterns (PAMPs) or damage-associated molecular patterns (DAMPs) from host origins bind to pattern recognition receptors (PRRs) to activate NLRP3. This activation recruits ASC, which binds to and hydrolyzes Caspase-1 precursors, cleaving IL-1β and IL-18 precursors, ultimately releasing IL-1β and IL-18 to exacerbate the inflammatory response (25). Additionally, activated Caspase-1 mediates cellular pyroptosis by activating GSDMD (26). Studies have shown that the survival rate of mice with brain hemorrhage significantly increases after Caspase-1 knockdown (27). In acute respiratory distress syndrome (ARDS), ART has been reported to reduce local inflammation induced by lipopolysaccharide by weakening the NLRP3/ASC/Caspase-1 signaling axis (28). In the central nervous system (CNS), a study indicated that ART effectively protects against neurological impairment by reducing LPS-induced CNS inflammation, although the expression of NLRP3/ASC/Caspase-1 was not detected (29). Based on these findings, we hypothesize that ART participates in the NLRP3 signaling pathway to protect neurological function after cerebral hemorrhage.

Microglia are the resident immune cells of the CNS and play a crucial role in the early inflammatory response following ICH (30,31). They have been extensively studied for their significant secretory functions. Inhibiting the release of pro-inflammatory factors from microglia after ICH is a key strategy for mitigating secondary damage (32-34). Pyroptosis, a form of programmed cell death characterized by the release of inflammatory factors, primarily affects neurons and microglia following ICH (35,36). In this study, we confirmed that ART primarily impacts NLRP3-mediated pyroptosis within microglia after ICH. Our *in vitro* experiments also demonstrated ART's inhibitory effect on microglial pyroptosis. To our knowledge, this is the first report showing that ART can suppress pyroptosis and the release of pro-inflammatory factors in microglia after ICH, significantly reducing brain edema and neuronal damage in mice.

Given the irreversible secondary damage to neurological function caused by the local inflammatory cascade response in the posterior striatum following cerebral hemorrhage, this study was conducted to investigate the potential of ART in reducing the inflammatory cascade in cerebral hemorrhage. Initially, the results demonstrated that ART significantly improved brain edema in experimental animals after cerebral hemorrhage and enhanced performance in animal cornering experiments and mNSS scores. Next, we examined the expression levels of TNF-α, IL-1β, and MPO in animals after ART treatment using western blotting and immunohistochemical staining. In parallel, we assessed the infiltration of microglia in the striatum of each group using immunofluorescence. The results revealed that ART significantly reduced neuroinflammation in animals 3 days after ICH. To explore the mechanism behind these observations, we investigated the expression levels of the NLRP3/ASC/Caspase-1 signaling axis in each group of animals. The results showed that administering 150 mg/kg of ART significantly reduced the expression of NLRP3/ASC/Caspase-1 following ICH. Additionally, immunofluorescence analysis revealed a significant decrease in NLRP3 expression within microglia. These findings help explain the observed reduction in local inflammatory infiltration in the striatum of ART-treated animals.

The significance of inflammation-mediated neuronal apoptosis after ICH is profound. Neurons affect the neurovascular unit through trophic support, modulating the integrity of the blood-brain barrier (37). Damage to the neurovascular unit after ICH leads to leakage of local proteins, which contributes to the formation of fibrous scars, activation of astrocytes, and the formation of a local glial scar, ultimately impairing neurological function (38). Studies have shown a correlation between early neuronal apoptosis after ICH and poor patient prognosis, characterized by reduced cortical thickness, loss of white matter integrity, and hippocampal atrophy (39). The NOR test and Morris water maze experiments supported these findings, showing that ART intervention significantly alleviated memory and learning deficits caused by ICH. Therefore, the inhibition of early inflammatory infiltration after ICH has long-lasting and profound effects.

One limitation of this study is that it only used male mice, and thus the potential effects of sex differences on the results were not explored. While the inflammatory response and neuroprotective mechanisms in experimental cerebral hemorrhage models have been studied predominantly in male animals, there is evidence suggesting sexual dimorphism in neurological responses to injury and inflammation. Female animals may exhibit different inflammatory profiles, potentially leading to variations in neuroinflammatory processes and recovery after cerebral hemorrhage. For example, female mice have been shown to have lower mortality and improved neurobehavioral outcomes compared to male mice following brain hemorrhage, possibly due to differences in hormonal influences and immune responses (40). Future studies should incorporate both male and female animals to further investigate how sex may modulate the neuroprotective effects of artemisinin and its impact on the NLRP3 signaling pathway. Exploring these sex differences would provide a more comprehensive understanding of the therapeutic potential of ART in treating cerebral hemorrhage.

## Conclusion

Overall, our study showed that ART attenuated microglia pyroptosis early after ICH by blocking the microglia NLRP3/ASC/Caspase-1 signaling pathway and ultimately reduced neuronal apoptosis and brain edema conditions to protect neurological function in experimental animals.

## Data Availability

The datasets generated and/or analyzed during the current study are available from the corresponding author on reasonable request.
